# Non-canonical *Drosophila X* chromosome dosage compensation and repressive topologically associated domains

**DOI:** 10.1186/s13072-018-0232-y

**Published:** 2018-10-24

**Authors:** Hangnoh Lee, Brian Oliver

**Affiliations:** 1 Laboratory of Cellular and Developmental Biology, National Institute of Diabetes and Kidney and Digestive Diseases, National Institutes of Health, Bethesda, MD USA; 2 Section on Cell Cycle Regulation, National Institute of Child Health and Human Development, National Institutes of Health, Bethesda, MD USA

**Keywords:** Dosage compensation, *Drosophila melanogaster*, MSL complex, Topologically associated domain, Lamina-associated domain

## Abstract

**Background:**

In animals with *XY* sex chromosomes, *X*-linked genes from a single *X* chromosome in males are imbalanced relative to autosomal genes. To minimize the impact of genic imbalance in male *Drosophila*, there is a dosage compensation complex (MSL) that equilibrates *X*-linked gene expression with the autosomes. There are other potential contributions to dosage compensation. Hemizygous autosomal genes located in repressive chromatin domains are often derepressed. If this homolog-dependent repression occurs on the *X*, which has no pairing partner, then derepression could contribute to male dosage compensation.

**Results:**

We asked whether different chromatin states or topological associations correlate with *X* chromosome dosage compensation, especially in regions with little MSL occupancy. Our analyses demonstrated that male *X* chromosome genes that are located in repressive chromatin states are depleted of MSL occupancy; however, they show dosage compensation. The genes in these repressive regions were also less sensitive to knockdown of MSL components.

**Conclusions:**

Our results suggest that this non-canonical dosage compensation is due to the same transacting derepression that occurs on autosomes. This mechanism would facilitate immediate compensation during the evolution of sex chromosomes from autosomes. This mechanism is similar to that of *C. elegans*, where enhanced recruitment of *X* chromosomes to the nuclear lamina dampens *X* chromosome expression as part of the dosage compensation response in *XX* individuals.

**Electronic supplementary material:**

The online version of this article (10.1186/s13072-018-0232-y) contains supplementary material, which is available to authorized users.

## Background

Genes come in pairs and large-scale deviation from this state is detrimental, most probably as a result of disrupted gene expression balance [1, 2]. Sex chromosomes are a peculiar exception to this general rule. In *XY* systems, males have what amounts to a heterozygous deletion of an entire chromosome, bearing ~ 20% of the genes in the case of *Drosophila*, with no impact on fitness. In such systems, compensation often rectifies gene dose effects as a way to maintain gene balance [3–5].

In *Drosophila melanogaster*, a male-specific complex called the Male-Specific Lethal (MSL) complex plays a role in equalizing expression of genes from the single *X* chromosome relative to autosomes. MSL and other unidentified sources of compensation ultimately achieve remarkably equalized levels of *X*-linked gene expression in males with one *X* and females with two *X*s, as well as balancing *X* expression with the autosomes [6–8]. The complex includes MSL-1, MSL-2 and MSL-3 proteins, Maleless (MLE), and Males absent on the first (MOF) proteins, and two noncoding RNAs, *roX1* and *roX2* [9]. MOF has a histone acetyltransferase activity and functions in enhanced elongation of *X* chromosome gene transcription by acetylating Histone H4K16 (H4K16Ac) [10]. There exist two different models that describe how the MSL complex achieves *X* dosage compensation [8, 11]. In one model, MSL has a positive role in upregulating the *X*-linked genes [5, 11]. The boosting of expression is primarily achieved via enhanced elongation of transcription [10], but there also is evidence that RNA polymerase II (Pol-II) binding is increased by 1.2-fold at male *X* chromosome promoters [12–14]. In the second model, *X* chromosome dosage compensation is mainly achieved by an inverse dosage effect; MSL proteins only have an indirect role by sequestering MOF to the male *X* chromosome to prevent over-expression of the genes [8, 15–17]. In both models, the molecular evidence demonstrates that MSL complex does not bind at each promoter [15, 18]. Binding of MSL complex to the male *X* chromosome occurs at chromosome entry sites (CES), also referred to as high-affinity sites (HAS) [18, 19]. The sites contain GA-rich sequences, called the MSL recognition element (MRE) [18].

There is abundant evidence that MSL does not explain all *X* chromosome dosage compensation by either the activation or sequestration models. For example, dosage compensation has been seen in the early embryo before the MSL complex is established [20] and *X* chromosome dosage compensation in the germ line occurs even though the MSL complex is not required in the germ line [21]. Even after dosage compensation is established, it has been suggested that parts of the *X* are compensated independently from the MSL complex [22]. Furthermore, in cases where MSL involvement in a gene dosage compensation is clear, there is quantitatively unexplained dosage compensation [23]. At least a part of such “missing” compensation is mediated by normal gene network functions such as feedback. In S2 cells this mechanism is very substantial [23], perhaps due to selection in the dish, but in other cell lines gene regulatory networks make a smaller contribution [24]. In whole animals, this type of dosage compensation is seen in flies heterozygous for multi-locus deletions [25–27]. This compensation is highly gene dependent, but overall there is still missing dosage compensation. We estimate that there is roughly 1.4-fold compensation from the MSL complex [23], 1.1-fold from gene regulation [25–27] and about 1.3- to 1.4-fold missing compensation.

Dosage compensation mechanisms in other organisms provide ideas for how additional non-canonical dosage compensation in *Drosophila* might be mediated. In *C. elegans*, *XX* worms are hermaphrodites and *X0* worms are males. In *X0* males, the yield of *X* chromosome gene products is increased using various mechanisms (e.g., increased Pol-II recruitment, mRNA stability, or translation rate) in both males and hermaphrodites [21, 28–30]. However, solving the gene production difference between autosomes and *X* chromosomes in males results in over-expression in *XX* animals. To manage this increased activity, *XX* hermaphrodite *C. elegans* has a dosage compensation complex that represses gene expression from both *X* chromosomes [5, 29]. The *C. elegans* dosage compensation complex (DCC) targets the *X* chromosomes and spreads from recruitment sites on the *X* [31]. Recruitment of DCC on *X* chromosome is linked to increased mono-methylation of Histone H4K20 (H4K20me1) [32, 33], as well as depletion of histone modifications that mark active transcription, such as H4K16Ac [29, 34, 35] and H2A.Z variant histone [36]. These epigenetic changes accompany topological remodeling of the *X* chromosomes [37] and reduced Pol-II recruitment at *X*-linked promoters in hermaphrodites. [3, 5, 38]. This remodeling includes nuclear sub-localization of the *X* chromosomes to the lamina, which is repressive. Disruption of the anchoring between heterochromatin and nuclear lamina re-localizes *X* chromosomes more centrally in the nucleus and results in partial derepression of the *X*-linked genes [39]. Thus, the modulation of H4K16Ac in animals with a single *X* is a conserved characteristic between *D. melanogaster* and *C. elegans* [34] although the *XX* mechanisms differ [35].

Intriguingly, the type of nuclear architecture-level derepression of the *C. elegans* X also occurs in autosomal dosage compensation in *D. melanogaster*. Genes within repressive “topologically associated domains” (TADs), which include lamina-associated domains (LADs), show better autosomal dosage compensation in *Drosophila* hemizygotes [25]. Unlike the gene-by-gene network effects also seen in these same hemizygotes, the deletions of LAD domains affect blocks of genes. The effect of autosomal deletions is derepression of the non-deleted genes *in trans*, as well as a spreading of derepression into flanking regions within the LAD. This suggests that these repressive domains are built based on additive or synergistic cooperation between gene homologs. Overall, in LAD regions derepression results in 1.1- to 1.2-fold dosage compensation, above the gene-by-gene effect of network interactions [25]. This observation is of particular interest for two reasons. First, the necessity of two homologs for the repression is reminiscent of chromosomal pairing-dependent events, such as pairing-sensitive silencing [40, 41] or transvection [42, 43]. In transvection, the existence of homologous chromosome in proximity leads to enhancer action *in trans* or insulator bypass *in cis* [42]. As such, chromosomal pairing may provide a mechanistic basis of how autosomal deletions result in the derepression of non-deleted genes [25]. The absence of a pairing partner for the single *X* in males might, therefore, be consequential. Second, the repression at the two-dose state, and derepression at one-dose state, is analogous to *X* chromosome dosage compensation in *C. elegans*. This led us to ask whether the derepression of one-dose genes from repressive domains occurs on *D. melanogaster X* chromosomes. If so, this would contribute to dosage compensation in males.

## Results

### X-linked repressive TADs genes display low expression levels, but are dosage compensated in males

**Fig. 1:**
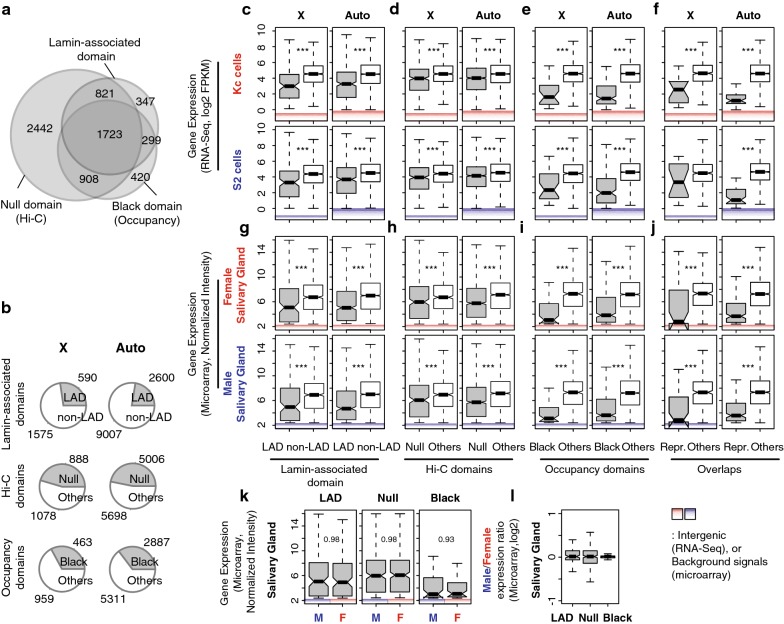
Repressive TAD genes display lower gene expression levels and are dosage compensated in male cells. **a** Venn diagram displays overlap among the three repressive TADs that are described in this study. **b** Pie charts demonstrate the proportion of repressive TAD genes (gray) versus non-repressive TAD genes (white) in *Drosophila* genome. In **a** and **b**, only protein-coding polyA^+^ genes are counted. The numbers do not directly indicate numbers of “expressed” genes in each TAD. **c**–**j** Gene expression levels from the repressive TAD genes (gray) and non-repressive TAD genes (white) based on LAD (**c**, **g**), Hi-C (**d**, **h**), chromatin occupancy studies (**e**, **i**) and their overlaps (**f**, **j**). The top two rows show RNA-Seq results from *Drosophila* cell lines (unit: log2 FPKM, **c**–**f**), and the bottom two rows are from a microarray study done with larval salivary glands (unit: normalized signal intensity, **e**–**j**). Intergenic signals from the 99th percentiles and below in RNA-Seq analyses, as well as background signals from the 99th percentiles and below in the microarray result, are indicated. **k** Comparisons of *X* chromosome gene expression levels from the repressive TADs between female and male salivary glands. **l** Comparisons of male *X* chromosome genes from the repressive TADs to the same gene in females. Boxplots indicate the distribution of gene expression levels above expression cutoffs. Middle lines in box display medians of each distribution. Top of the box. 75th percentile. Bottom of the box. 25th percentile. Whiskers indicate the maximum, or minimum, observation within 1.5 times of the box height from the top, or the bottom of the box, respectively. Notches show 95% confidence interval for the medians. ***p < 0.001 from Mann–Whitney *U* test. The same format and test have been used for all boxplots appeared in this study

To determine the overall structure of chromatin domains on the *X*, we used results from three previous studies that divided the genome into repressive versus non-repressive chromatin domains/TADs and LADs versus non-LADs. LAD and DamID (DNA adenine methyltransferase identification)-based chromatin occupancy information was from *Kc* cells [44, 45]. TAD information was from Hi-C conformation capture from mixed sex embryos [46]. From the Hi-C study, “Null” TADs were characterized by general lack of chromatin marks, except for a weakly enriched binding of an insulator protein, Suppressor of Hairy-wing [SU(HW)]. The LAD and “Null” TADs correspond and largely overlap with “Black” domain DamID work. The “Black” domain has increased signals of Histone H1, Effete (EFF), Suppressor of Under-Replication (SUUR) and Lamin B protein binding. These repressive TADs are known to share various characteristics [46], and there are significant overlaps among the identified gene sets (Fig. 1a, Table 1). For example, 63% of genes that are in LADs are also in Black domains, and 79% of genes that are in Black domains are in Null domains. We collectively refer to these overlapping domains as “repressive TADs.” Gene ontology analysis indicated developmental stage, or tissue, specific functions of the repressive TAD genes (Additional file 1).

**Table 1:** Number of genes expressed in repressive TAD classes

Domains	# Genes		# Expressed in Kc cells		# Expressed in S2 cells		# Expressed in female salivary glands		# Expressed in male salivary glands	
	*X*	Auto	*X*	Auto	*X*	Auto	*X*	Auto	*X*	Auto
LAD	590	2600	113 (8.16)^a^	350 (9.74)	126 (9.54)	382 (12.71)	110 (5.29)	507 (4.93)	110 (5.25)	503 (4.66)
Null	888	5006	352 (15.82)	1633 (16.17)	346 (15.43)	1593 (17.89)	278 (6.03)	1465 (5.78)	276 (6.14)	1460 (5.74)
Black	463	2887	26 (3.06)	145 (2.75)	32 (5.08)	155 (3.85)	52 (3.06)	369 (3.77)	52 (3.13)	372 (3.61)
Repressive TADs (overlap)	285	1438	16 (5.99)	41 (2.22)	17 (10.22)	47 (2.09)	30 (2.80)	165 (3.64)	31 (2.84)	165 (3.53)
Non-repressive TADs (overlap)	621	3411	545 (24.75)	2725 (24.22)	535 (22.78)	2623 (25.12)	376 (7.34)	1863 (7.26)	377 (7.32)	1865 (7.34)

^a^Median gene expression levels in FPKMs (*Kc* and S2 cells) and normalized intensity (salivary glands)

Each of these three repressive TADs covered 23 to 43% of the protein-coding genes in the *Drosophila* genome. To describe which genes on the *X* were in repressive TADs, we parsed by chromosome (Fig. 1b). Collectively, genes within LADs included 27% of *X* chromosome genes and 22% of autosomal genes (*p* = 0.00015, Fisher’s exact test, protein coding only). Genes within Null domains included 41% of *X* chromosome genes and 43% of autosomal genes (*p* = 0.25). Genes within Black domains included 21% of *X* chromosome genes and 25% of autosomal genes (*p* = 0.0059). Clearly, a large fraction of the genome, including the *X*, are in repressive domains. If these genes are simply “off,” then asking whether they are dosage compensated is a futile effort (2 × 0 = 0). Therefore, we carefully examined expression levels from genes that are within the repressive domains to see whether we could reliably detect expression. We used previously reported expression data for this analysis [47, 48]. Expression levels in repressive domains were reassuringly lower than in non-repressive domains. We found these trends of lower expression in repressive TAD genes when we investigated different cell lines (Fig. 1c–f) and sexed salivary glands (Fig. 1g–j), but there was clear evidence of expression.

Determining the difference between low and off is critical for this analysis. We measured the biological and technical noise levels by measuring intergenic signals (Fig. 1c–f). The 99th percentiles for intergenic signal were 0.87 Fragments Per Kilobase of transcript per Million mapped reads (FPKM) in S2 cells (male) and 0.98 FPKM in *Kc* cells (female). This is in stark contrast to expression levels in the repressive TADs from *Kc* cells, where LAD and Black domains were determined (Fig. 1c–f, the top panel). The median *X*-linked gene expression level was 8.2 FPKM for genes within LADs and 15.8 FPKM for genes within Null domains in *Kc* cells. Genes in Black domains showed lower expression at a median of 3.1 FPKM, but all of these expression levels far exceed our estimates of noise. In *Kc* cells, approximately 19.2% and 39.6% of the *X*-linked genes demonstrate gene expression above the cutoff levels from LAD and Null domains, respectively (Table 1). Only 5.6% of the *X*-linked genes were expressed from Black domains, indicating that the Black domain has the most repressive characteristics among three different calls of repressive TADs. Autosomal genes from repressive TADs also displayed lower gene expression levels compared to non-repressive TAD genes with 9.7 (LAD), 16.1 (Null) and 2.8 FPKMs (Black), which are not significantly different from repressive TAD genes on the *X* (*p* > 0.2, Mann–Whitney *U* test). In male S2 cells, the repressive TAD genes demonstrated 9.5 (LAD), 15.4 (Null) and 5.1 FPKMs (Black) on the *X* chromosome. We made a similar observation from sexed salivary glands. A large fraction of genes from repressive TADs showed expression higher than technical noise, which we determined based on background signals from the control probes of microarrays (Fig. 1g–j, normalized intensities of approximately 2.4 in both sexes). For example, about 18.6% of the total *X*-linked LAD genes showed gene expression above the background levels in both female and male salivary glands. Thus, we were confident that a substantial portion of the genes in repressive TAD domains showed detectable levels of gene expression. We used these genes in our analysis.

Genes in repressive TADs demonstrated comparable expression levels between female (*Kc*) and male (*S2*) cells from the *X* (Fig. 1c–f), indicating that they are dosage compensated. However, both *S2* and *Kc* cells are highly aneuploidy [48], and S2 cells show very pronounced gene-by-gene network-mediated dosage compensation [23]; thus, they are not the best models for determining whether some *X* chromosome dosage compensation occurs by derepression. Therefore, we also compared expression profiles from salivary glands from female and male siblings, to analyze *X*-linked gene expression in repressive TADs. From microarray results, we observed that male *X*-linked genes from LAD regions demonstrated comparable expression levels to those of females (Fig. 1k). The median signal intensity from male *X*-linked genes was 5.25, which did not differ from that of female (5.29, *p* = 0.984) despite the 50% difference in *X* gene dose. We obtained similar equilibrated expression of the *X* from other repressive TADs. *X* chromosome genes in the Null domains showed a median of 6.14 signal intensity in *X* males when it was 6.03 in *XX* females. Black domain genes had medians of 3.06 and 3.13 signal intensities in *X* males and *XX* females, respectively. Overall gene expression signals from autosomes were consistent between two sexes (6.82, *p* > 0.819 for differential expression). Therefore, the repressive TAD genes are dosage compensated in males. When we compared expression levels of each gene in females directly to those of males, we obtained log2 ratio closed to 0 from all different repressive TAD classes (Fig. 1l).

### Repressive TAD genes lack MSL complex binding

**Fig. 2:**
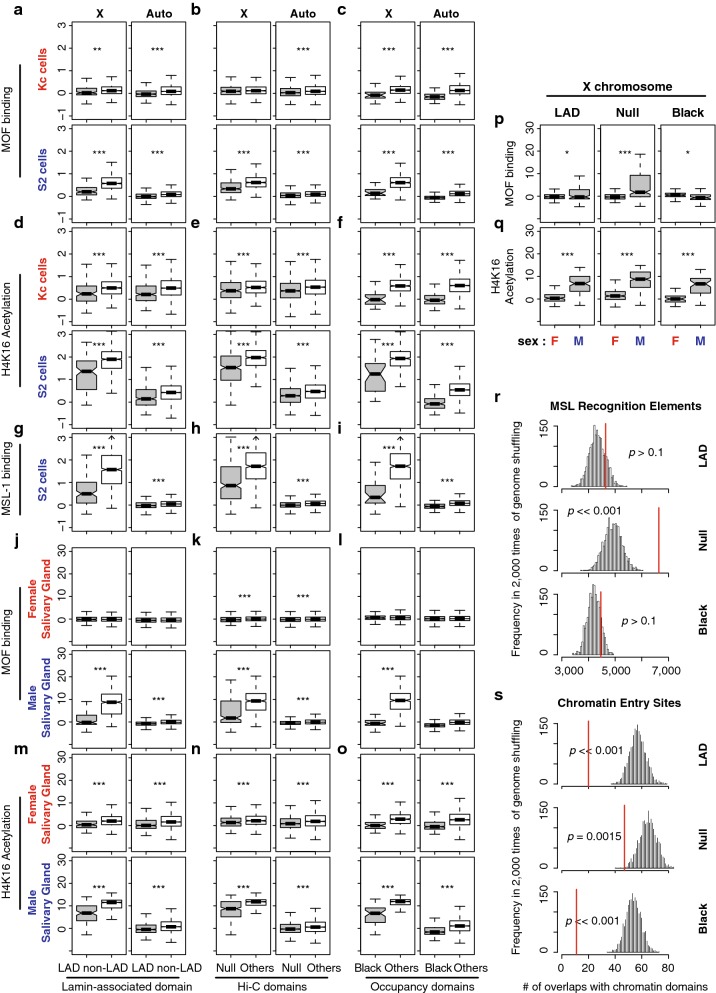
Repressive TAD genes have a limited binding of MSL complex. **a**–**i** Chromatin immunoprecipitation (ChIP) results from MOF binding (**a**–**c**), Histone H4K16 acetylation (**d**–**f**) and MSL-1 binding (**g**–**i**) are summarized as boxplots for *Drosophila* cell lines (*Kc* and *S2*). Gene-level ChIP signals are separately shown based on LAD (**a**, **d**, **g**), Hi-C (**b**, **e**, **h**) and chromatin occupancy (**c**, **f**, **i**) study results. **j**–**o** ChIP results from the third instar larval salivary glands. **p < 0.01, ***p < 0.001. No asterisk indicates *p* > 0.05. **p**, **q** Direct comparisons of MOF binding (**p**) and H4K16Ac enrichment (**q**) between female and male salivary glands from **j**–**o**. **r**, **s** The histogram represents expected numbers of overlaps between repressive TADs and MRE (**r**), or CES (**s**). We performed random shuffling of the *X* chromosome genome 2000 times and demonstrated the frequencies of the number of overlaps. Red lines: the actual number of overlaps between LADs and MREs or CES’s. *p* values are from permutation tests

Our hypothesis is that *X* specific dosage compensation has canonical and non-canonical components. If canonical dosage compensation is active in repressive domains, the MSL complex should occupy those regions. To address this possibility, we first investigated chromatin occupancy by MOF, the key writer of the H4K16Ac mark [49] in the MSL complex [9]. MOF also has an MSL-independent role in regulating a smaller subset of genes in both sexes by participating in non-specific lethal (NSL) complex [50]. We analyzed genome-wide chromatin immunoprecipitation (ChIP) results [47, 51] to determine occupancy of the MSL complex as well as H4K16Ac levels in tissue culture cells and salivary glands (we measured MOF and H4K16Ac enrichment within gene bodies because both MOF and H4K16Ac display broad enrichment patterns over these features [50]). Strikingly, in male S2 cells, MOF binding in *X* chromosome repressive TADs was significantly lower than elsewhere on the *X* (*p* < 6.01e−4, Fig. 2a–c). H4K16Ac enrichment concurred with MOF occupancy. In all classes of *X* chromosome repressive TADs, H4K16Ac levels were significantly lower than in other domains (*p* < 6.96e−09, Fig. 2d–f). In S2 cells, H4K16Ac levels on *X*-linked genes were still higher than those of autosomal genes even within repressive TADs (*p* < 4.92e−15), which was not the case in *Kc* cells (*p* > 0.57). MSL complex preferentially targets active genes with H3K36me3 marks [52]. Consistently, we found that genes within repressive TADs show significantly lower H3K36me3 levels than genes from the non-repressive TADs in S2 cells (*p* < 1.0e−08, Additional file 2). Additionally, we observed that another MSL component, MSL-1, showed lower occupancy in genes within repressive TADs on the *X* compared to non-repressive TADs (*p* < 1.11e−12, Fig. 2g–i). Thus, the occupancy and activity of MSL complex were reduced in the case of the dosage-compensated *X*-linked genes in *S2* cell repressive TADs.

To examine MSL complex activity at the repressive TADs in tissues, we analyzed ChIP results from sexed larval salivary glands. In males, X chromosome MOF binding was significantly higher at gene bodies in non-repressive TADs, compared to repressive TADs (Fig. 2j–l, *p* < 2.2e−16). If MOF binding is functional, then the H4K16Ac mark should follow a matching enrichment pattern. Indeed, H4K16Ac levels were higher at genes in non-repressive TADs compared to repressive TADs (Fig. 2m–o, *p* < 2.2e−16). The basal level of MOF binding and H4K16Ac was higher in both repressive and non-repressive TAD genes of the male salivary glands, compared to that of female glands (*p* < 0.046 for MOF and *p* < 2.2e−11 for H4K16Ac, Fig. 2p, q). However, the differences in MOF binding and H4K16Ac levels between male and female cells were significantly smaller in LAD and Null domains than non-repressive TADs on the *X* (*p* < 2.0e−05 for both MOF binding and H4K16Ac, permutation test). This observation indicates that regulation of repressive TAD genes on the *X* chromosome occurs with limited or transient access to MSL complex, but this also suggests that repressive TADs might also use modulations of H4K16Ac in a canonical manner. Our result is consistent with a previous study that showed the exclusive positioning of MSL complex in the active compartment of the nucleus [53].

Since the genes within repressive TADs have low occupancy of MSL complex and lower H4K16Ac, we wondered whether repressive TADs lack genomic signatures that are required for MSL complex binding. Specifically, we asked whether lower MOF activity correlates with the lower density of the MSL complex entry sites in repressive domains. *Drosophila* MSL complex specifically binds to *X*, which occurs at CES [18]. CES contains GA-rich DNA sequence motif, called MRE, whose introduction to an autosome resulted in local recruitment of MSL complex to that site [18]. We identified 11,306 MRE motifs from the *X* chromosome of the reference genome (using an *E* value < 10e−5 cutoff). The number of *X* chromosome MREs in repressive domains was not statistically different from random (Fig. 2r, *p* > 0.1 permutation test), indicating that the repressive TADs are not free of MRE motifs. However, when we investigated whether genes in repressive TADs recruit MSL complex to their chromatin regions, we found only 20 overlaps between LADs and the 150 CES (approximately 57 expected, *p* ≪ 0.001, permutation test, Fig. 2s) that recruit MSL [18]. We obtained consistent results from Null and Black domains (Fig. 2r, s). These observations suggest that on male *X* chromosomes, MSL complex does not efficiently bind genes within the repressive TADs.

**Fig. 3:**
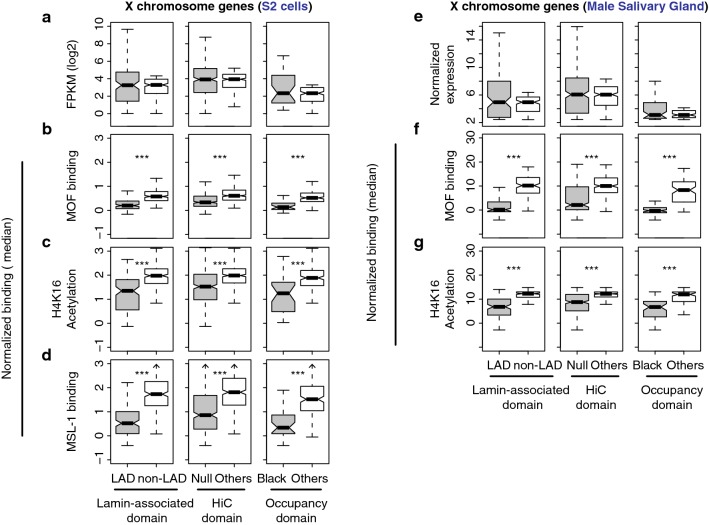
Lower occupancy of MOF and H4K16Ac levels in the *X*-linked genes within repressive TAD compared to non-repressive TAD genes with similar expression levels. **a** Boxplots display gene expression levels from S2 cells in FPKMs. The *X*-linked repressive TAD genes (gray) and genes from non-repressive TADs (white) were compared; for the latter, highly expressed genes were removed to equalize the medians. **b**–**c** MOF occupancy, H4K16Ac level or MSL-1 binding of the genes in (**a**). **e** *X* chromosome gene expression levels from male salivary glands where the median expression of non-repressive TAD genes is matched to that of the repressive TAD genes as in (**a**). **f**, **g** MOF occupancy and H4K16Ac levels for the genes in (**e**)

H4K16Ac and MOF binding are related to expression levels, so it was possible that lowly expressed genes are compensated by MSL, with lower modification levels and occupancy simply because they have low expression levels. To test this possibility, we compared male *X* chromosome genes from the repressive TADs to non-repressive TAD genes that have similar low expression levels. We achieved this by filtering out highly expressed genes within non-repressive TADs to match non-repressive TAD expression medians to those of the repressive TAD genes (Fig. 3a). We observed that MOF binding was still significantly more enriched at non-repressive TAD genes, compared to the genes from repressive TAD classes (*p* < 6.94e−11, Mann–Whitney *U* test, Fig. 3b). Similarly, H4K16 acetylation level, as well as MSL-1 binding, was higher from the non-repressive TAD genes on the *X* chromosome, compared to the genes within the repressive TADs (*p* < 1.87e−07). We obtained consistent results from the male salivary glands (Fig. 3e–g). The genes within repressive TADs displayed significantly less MOF binding and H4K16Ac than the non-repressive TAD genes even when their expression medians were matched (*p* < 1.25e−12). Therefore, the lack of MOF binding and lower H4K16Ac levels in the *X*-linked repressive TAD genes are not simply due to their lower expression levels, suggesting that the activities of MSL complex are limited in repressive domains.

### X-linked repressive TADs genes are less sensitive to disruption of MSL-complex functions compared to the canonical dosage-compensation target genes

**Fig. 4:**
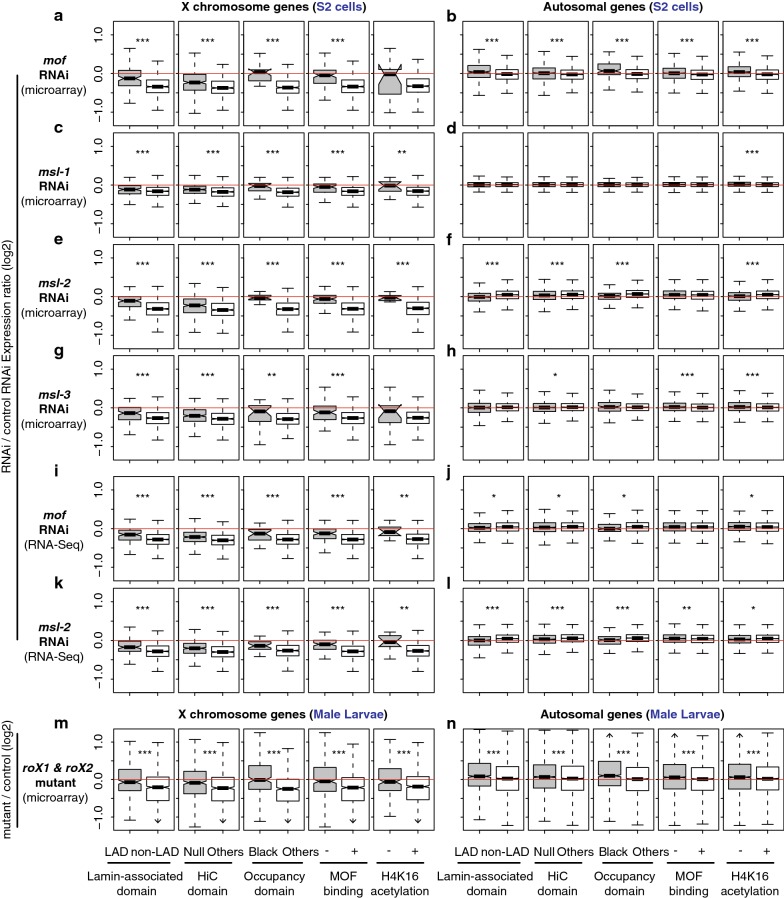
Different responses from the repressive versus non-repressive TAD genes upon knockdown or mutation of MSL complex components. Boxplots represent gene expression changes in log2 scale from depletion of MSL components in *Drosophila* S2 cells (**a**–**l**) or *roX* mutation in male larvae (**m**–**n**). Plots are based on four independent studies [23, 47, 54, 88], which used either microarray (**a**–**h** and **m**–**n**) or RNA-Seq technology (**i**–**l**). **a**, **b** Differential gene expression from *mof* knockdown cells. Changes from the repressive TADs (left three columns, LAD, Null and Black) as well as MOF binding, or Histone H4K16 acetylation regions are presented. **c–h** Results from *msl*-*1*, *msl*-*2*, or *msl*-*3* knockdown. **i**, **j** Results from *mof* knockdown, measured by RNA-Seq analysis. **k**, **l** *msl*-*2* knockdown. **m**, **n** Results from *roX1* and *roX2* null mutant larvae, measured by microarrays. **a**, **c**, **e**, **g**, **i**, **k**, **m** Changes from *X* chromosome genes. **b**, **d**, **f**, **h**, **j**, **l**, **n** Changes from autosomal genes. *p < 0.05, **p < 0.01, ***p < 0.001

If the repressive TAD genes are dosage compensated in a non-canonical way on the male *X* chromosome, such genes might be indifferent to MSL complex function. In contrast, if the low level of H4K16Ac is matched to the low-level expression of the genes in repressive domains, compensation of such genes should depend on MSL function. To investigate the impact of disrupted MSL complex function on *X*-linked genes in repressive TAD domains, we analyzed gene expression profiles of S2 cells whose MSL components were selectively depleted via RNAi-mediated knockdown [23, 47, 54]. When *mof* mRNA was depleted, *X*-linked genes within LADs were significantly less sensitive to MOF reduction than genes in non-LAD domains (*p* = 1.1e−13, Mann–Whitney *U* test, Fig. 4a). We made similar observations from *X* chromosome genes that belong to Null and Black domains from the Hi-C study and occupancy study. They exhibited higher relative expression upon the depletion of *mof* than other *X*-linked genes in non-repressive domains (*p* < 2.3e−08). As expected, those chromatin regions that lack MOF binding and H4K16Ac were less sensitive to the RNAi treatment as well (*p* = 1.1e−14 for MOF and 0.11 for the acetylation). MOF is also bound to sites on autosomes as a part of NSL complex, while it activates only a small subset of genes that the complex binds to [55]. Consistent with this idea, we saw little down-regulation of overall autosomal gene expression from the *mof* depleted S2 cells (*p* > 0.05, Fig. 4b).

We also asked whether the expression of *X*-linked genes in repressive TADs was less sensitive to depletion of other MSL components. Our analysis showed significantly less reduction in expression in the gene within repressive TADs, relative to non-repressive TADs, when *msl*-*1* mRNA was depleted (Fig. 4c, d, *p* < 0.001). Similarly, *msl*-*2* and *msl*-*3* knockdown caused more *X* chromosome gene expression from genes in repressive TADs, compared to non-repressive TADs (Fig. 4e–h, *p* < 0.01). These results were not due to the inaccurate detection of low-abundant transcripts in hybridization-based techniques (i.e., microarrays) [56, 57]. When we analyzed an independent study that performed RNA-Seq analysis of either *mof* or *msl*-*2* depleted S2 cells, we also observed about more expression from the *X*-linked genes within repressive TADs compared to non-repressive TADs (*p* < 0.001, Fig. 4i–l). Supporting the idea from RNAi experiments, *Drosophila* male larvae that are null for noncoding RNA components of MSL complex (*roX1* and *roX2*) demonstrated more expression from X-linked genes in repressive TADs compared to genes in non-repressive TADs (*p* < 5.56e−07, Fig. 4m, n). Collectively, our results from the MSL inhibition were consistent with our observation in Fig. 2 that demonstrated limited occupancy of MSL complex at repressive TAD genes, and suggest that genes in repressive TADs on the *X* chromosome do not rely entirely on MSL complex for dosage compensation.

**Fig. 5:**
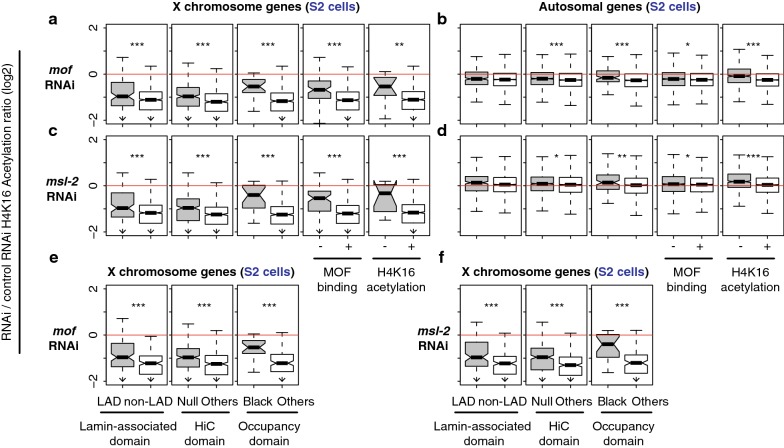
H4K16Ac levels change less within the repressive TADs when MSL components are depleted. Boxplots show changes in H4K16Ac levels upon the RNAi-mediated knockdown of *mof* (**a**–**b**) or *msl*-*2* (**c**–**d**). **a**, **b** Results from *mof* knockdown. **c**, **d** Results from *msl*-*2* knockdown. **e**, **f** Changes in H4K16Ac as in A and B, but highly expressed genes within non-repressive TADs were filtered out to match their expression median to that of the repressive TAD genes as in Fig. 3. **a**, **c**, **e**, **f** Results from X-linked genes. **b**, **d** Results from autosomal genes

We investigated whether the insensitivity to *msl* knockdown is also reflected in H4K16Ac levels in repressive TADs. We re-analyzed ChIP-chip results from S2 cells [23]. Consistent with the observation of gene expression changes, RNAi-based depletion of *mof* and *msl*-*2* had less impact on H4K16Ac levels of the *X*-linked genes within repressive TADs than non-repressive TADs (Fig. 5a–d, *p* < 0.0038). The smaller change of H4K16Ac upon the RNAi from the repressive TADs was not due to the low expression or H4K16Ac levels of the genes. When we compared median-matched gene expression from repressive versus non-repressive TADs (Fig. 3a), we still observed that H4K16Ac levels in repressive TADs were significantly less sensitive to *mof* and *msl*-*2* knockdown (Fig. 5e, f, *p* < 6.48e−06). MOF is the writer of the H4K16Ac mark, so this result is surprising. Perhaps, H4K16Ac marks in repressive domains are more resistant to conversion by histone deacetylases, or additional histone acetyltransferases may still function in the domains.

**Fig. 6:**
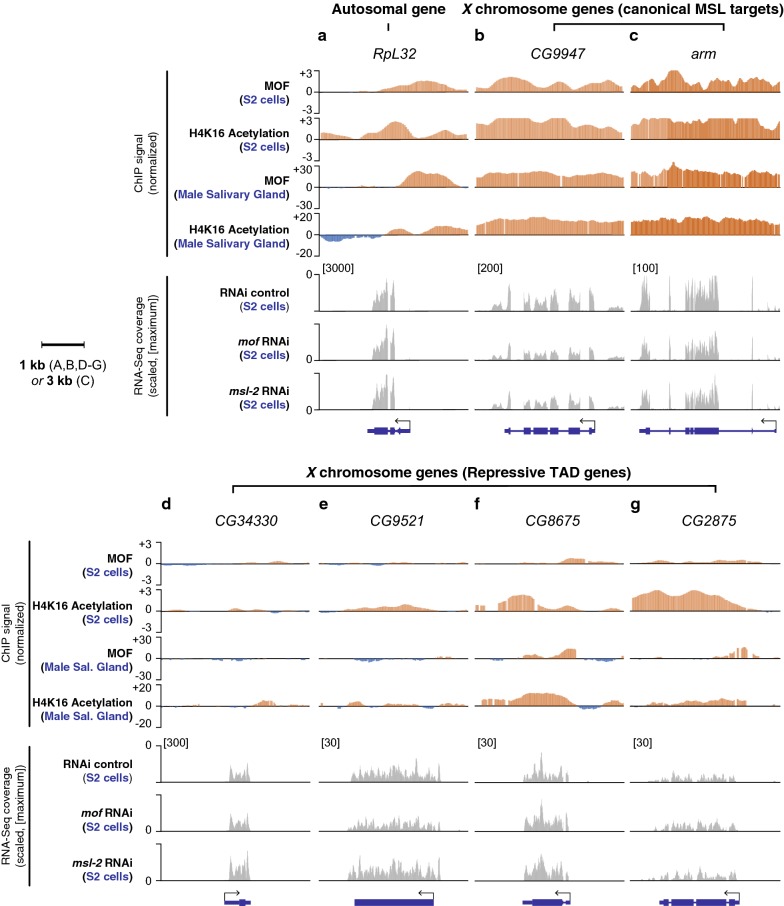
Repressive TAD genes that are less sensitive to *msl* or *mof* knockdown lack MOF enrichment at their gene bodies. Top four panels demonstrate normalized ChIP signals of MOF and H4K16Ac from S2 cells as well as male salivary glands. The bottom three panels display RNA-Seq read coverages from our re-analysis of Zhang et al. [23]. The plots are scaled based on their maximum coverage from one of the three samples: control RNAi, *mof* RNAi and *msl*-*2* RNAi (indicated in the square brackets). Note that there is no sample-to-sample normalization because the total number of reads is similar across the three samples; 7.2, 7.5 and 8.1 million mapped reads for the control, *mof* RNAi and *msl*-*2* RNAi samples, respectively. **a** An autosomal gene (*RpL32*). **b**, **c** Canonical MSL targets genes. *CG9947* (B) and *arm* (**c**). **d**–**e** Repressive target genes that are compensated via the non-canonical dosage compensation. *CG34430* (**d**), *CG9521* (**e**), *CG8675* (**f**) and *CG2875* (**g**)

The patterns of MOF occupancy and H4K16Ac differ between autosomes and the *X* [50]; therefore, investigating their patterns for individual genes might help inform the role of MSL in repressive TADs. We observed genes that were sensitive to the *msl* or *mof* knockdown, for example, *CG9947* and *arm*, which had broad ChIP signals of MOF and H4K16Ac in contrast to an autosomal gene, *RpL32*, which has MOF enrichment only at its promoter region (Fig. 6a–c). Compared to canonical MSL target genes, the genes in repressive TAD regions showed absent MOF binding (Fig. 6d, e, *CG34330* and *CG9521*), or weak MOF occupancy (Fig. 6f, g, *CG8675* and *CG2875*). In all four specific cases, the knockdown of *mof* or *msl*-*2* did not lead to statistically significant reduction of gene expression in males (*p* > 0.7, Fig. 6d–g); additionally, the genes were still fully compensated relative to females in the salivary glands [male/female expression ratios of 1.02 (*CG34330*), 1.02 (*CG9521*), 1.04 (*CG8675*) and 0.97 (*CG2875*)]. For the latter class of genes that have weak MOF occupancy (*CG8675* and *CG2875*), we noticed that MOF also bound at the 3′ ends of genes and H4K16Ac signal has additional peaks at the 3′ ends. Genes that were clearly regulated by the canonical dosage compensation machinery (i.e., MSL dependent) display broad enrichment signals of MOF and H4K16Ac across the gene body regions, whereas MSL-independent MOF target genes (e.g., MOFs in NSL complex) show promoter-enriched MOF binding patterns [50]. Therefore, MOF and H4K16 enrichments at 3′ end of CG8675 and CG2875 indicate that there was some residual MSL activity for *CG8675* and *CG2875*, rather than NSL, in addition to the non-canonical dosage compensation mechanisms.

## Discussion

### Accounting for dosage compensation

Dosage expression responses as a result of autosome or *X* chromosome deletions, or the gross aneuploidy in tissue-culture cell lines [21, 23–27, 58–60], raise the possibility that full twofold *X* chromosome dosage compensation would be achieved via different layers of mechanisms. We hypothesize that there are gene-by-gene regulatory responses, regional responses and chromosome-wide responses. While one should be careful not to take precise fold changes too literally as compensation varies by gene and region, some basic accounting illustrates these layers. Gene-by-gene regulation can account for 1.1-fold upregulation of dosage compensation, based on comparing one- to two-dose gene expression values on the autosomes or on the *X* in females [25, 26], leaving other dosage compensation mechanisms a 1.8-fold task on average. Regional derepression can account for 1.1- to 1.2-fold upregulation in genes within repressive TADs [25], although in some cases this is greater than twofold on the autosomes. We hypothesize that in dosage-compensated regions of the *X* where the MSL complex has a limited access [22, 53], derepression-based compensation may be the major contributor to compensation. The combination of gene-by-gene compensation and derepression leaves MSL complex, and other unknown mechanisms, with about 1.5-fold task in those regions for the full compensation. In this hypothesis, the major driving force of dosage compensation would be the MSL complex, but for each fully compensated X-linked gene in male fruit flies, there is a potential role for the gene network relationships as well as TAD nuclear architecture.

**Fig. 7:**
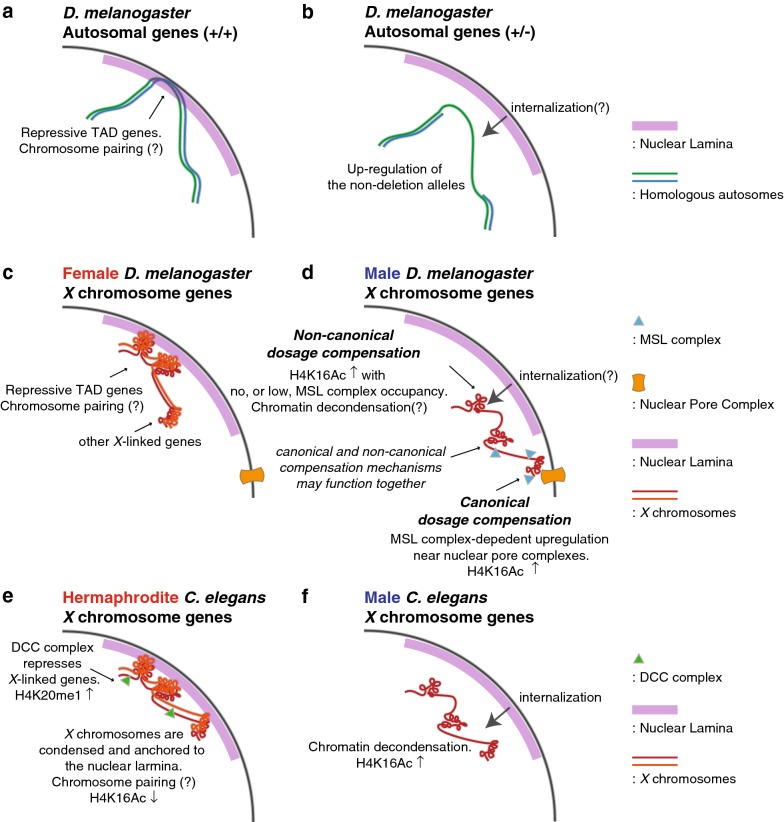
Hypothetical models demonstrating the parallelism among dosage compensation of autosomal dosage compensation in hemizygous *D. melanogaster*, and *X* chromosome dosage compensation in *C. elegans* and *D. melanogaster*. **a**, **b** A proposal of derepression-mediated compensation of one-dose autosomal genes in hemizygous *D. melanogaster* based on our previous study [25]. **c**, **d** A model of *X* chromosome dosage compensation in **d**. melanogaster based on the current study as well as other references [5, 7, 18, 50, 54, 91–93]. **e**, **f** A model of *X* chromosome dosage compensation in *C. elegans* based on the references [29, 32–35, 38, 39, 94]

In this work, we focused on regional non-canonical compensation within the boundaries of repressive TADs. On autosomes, deletions disrupting repressive TADs have a transacting derepressing effect on the hemizygous region [25], which results in partial dosage compensation for the hemizygous segment and over-expression of genes in flanking two-dose regions (Fig. 7a, b). These data suggest that repressive domains are established, strengthened, or stabilized by the existence of homologous pairs of chromosomes. There is strong precedent for pairing-dependent mechanisms in *D. melanogaster* that are known to activate or repress genes when homologous chromosomes are proximally located [40–43]. We propose a hypothesis that the unpaired *X* chromosomes of males have weaker repressive domains than the same domains in the paired *X* chromosomes of females (Fig. 7c, d). Thus, one can think of this as dosage compensation mediated by partial *X* inactivation in females, with derepression in males. This model hinges on the reorganization of the nuclear lamina–DNA interaction, which can clearly regulate gene activities during cell differentiation even in the absence of global changes of the nuclear architecture [61]. For example, in mouse embryonic stem cells, loss of the tethering in the *Hdac3* deletion releases genomic regions of lineage-specific genes from nuclear lamina resulting in precocious expression of those genes [62]. Tests for this hypothesis include systematically studying the effect of deletions of repressive TADs in females, which should result in partial dosage compensation like seen in hemizygous males, and analysis of chromatin structure differences between the sexes. Direct experiments on compartmentalization between the nuclear lamina and more centrally will be especially important.

### Evolutionary implications for non-canonical dosage compensation

Derepression of one-dose genes in *Drosophila* males is reminiscent of the *C. elegans* dosage compensation mechanism (Fig. 7e, f). In *C. elegans*, *XX* individuals are hermaphrodites and *XO* individuals are males. Both *X* chromosomes in hermaphrodites are subjected to dosage compensation control by repression [3, 5, 29]. The process involves DCC complex-dependent chromatin remodeling in *XX* hermaphrodites [32–34] that includes enrichment for H4K20me1 and depletion for H4K16Ac. In *X0* worms, the *X* shows de-condensation [35]. In addition to the chromatin remodeling, there is the local positioning of both *X* chromosomes of hermaphrodites to the LADs at the nuclear periphery which contributes to the repression of *X*-linked gene expression; the loss of this tethering results in derepression of *X*-linked genes in hermaphrodites [39]. The derepression of *X*-linked genes in tethering mutants of *cec*-*4* or *lem*-*2*, which encode a chromodomain protein or a component of nuclear lamina, respectively, results in a less extreme compensation phenotype than DCC mutants, raising the possibility that tethering to the nuclear lamina is an additional or supplemental mechanism to achieve dosage compensation by repression in *XX* individuals [39]. Thematically, this is identical to the non-canonical hypothesis for *Drosophila* dosage compensation that we propose to investigate.

Dosage compensation by derepression has interesting evolutionary implications. Specifically, we suggest that *X* chromosome dosage compensation by derepression relies on a general feature of repressive domains, requiring very little evolutionary innovation. As sex chromosomes evolve from an autosomal pair, the sex chromosome specific to the heterogametic sex becomes recombinationally silent and accumulates inversions, insertions and pseudogenes that further disrupt pairing [63–65]. As this process occurs, partial dosage compensation by derepression would be an immediate response, not requiring the evolution of any specific machinery. Improved dosage compensation can evolve to boost gene expression in *XY* males, by enhancing repression in *XX* females, or a combination of the two. This could account for some of the commonality between *D. melanogaster* and *C. elegans* dosage compensation mechanisms despite their divergence ~ 1 billion of years ago [66, 67]. In *Drosophila*, the *X* is specifically upregulated relative to autosomes in males [30] and is slightly overexpressed in females [68]. In *C. elegans*, the *X* is upregulated and is repressed specifically in hermaphrodites [4, 30]. Both these superficially divergent mechanisms could evolve from the same founding principles.

It has also been suggested that MSL drives the evolutionary content of the *X* chromosome, but our hypothesis of derepression makes the distribution of gene content on the *X* more explainable. There is a clear depletion of genes with male-biased expression in regions of high MSL occupancy, leading to the idea that MSL and increased expression drive these genes to new locations [69]. However, most of this male-biased expression occurs in the germ line. MSL complex does not function specifically on the *X* chromosome in the male germ line of *D. melanogaster* [70, 71]. The suggestion that MSL drives these genes to other locations seems spurious. We have shown that the regions without MSL entries sites correspond to the repressive TADs. Thus, we propose that *X*-linked genes with male germ line functions are more likely to be in repressive TADs, where they can show increased expression as a result of derepression. Indeed, in our previous results from gene expression profiling of hemizygote files with autosomal deletions [25], we observed that genes with male-biased expression in spermatocytes are derepressed in females when those repressive TADs are disrupted by deletions. There has been strong evolutionary pressure to relocate genes with male germ line function off the *X* chromosomes [72–74]. Those that remain might use derepression to achieve high expression even on the single *X*.

## Conclusion

We suggest the hypothesis that MSL complex-independent *X* chromosome dosage compensation exists in *Drosophila melanogaster*. We suggest that this non-canonical dosage compensation mechanism involves regional derepression of one-dose *X* chromosome genes in males, which are repressed in their two-dose state in females. We further suggest that this mechanism works to compliment gene-by-gene regulation and the chromosome-wide effects of MSL. This hypothesis has implications for the *X* chromosome dosage compensation evolution in systems where chromosome-wide mechanisms are active in either sex, as well as for evolution of gene content on the *X* in *Drosophila*.

## Materials and methods

### TADs information used in this study

We obtained LAD information from [44], HiC domains from [46] and DamID-based chromatin domains from [45]. All these results were generated based on *Drosophila* reference genome release 5. We used Flybase 5.57 gene model [75] in describing genes within such TADs. We defined genes to belong to TADs only when both boundaries of a gene are located in a TAD region. We performed our gene ontology analysis in FlyMine version 45.1 [76]. Results in the Additional file 1 represents significantly enriched terms, adjusted *p* value < 0.05, after Holm-Bonferroni correction.

### *Drosophila* cell line data from modENCODE studies

We used our previous results on RNA-Seq expression profiles of *Drosophila Kc* and S2 cells [48] for this study after updating gene IDs to FlyBase 5.57. We used FPKM > 1 as an expression cutoff based on the top 99th percentile of the intergenic FPKM signals (0.87 and 0.98 for *Kc* and S2 cells, respectively). We used the following chromatin immunoprecipitation (ChIP)-on-chip results from modENCODE study (model organism ENcyclopedia of DNA Elements) [51]. modENCODE submission IDs 3043 and 3044 for MOF binding in *Kc* and S2 cells, respectively, ID 318 for Histone H4K16 acetylation in *Kc* cells, IDs 319 and 320 for H4K16Ac in S2 cells, IDs 303 and 3170 for H3K36me3 in S2 cells and ID 307 for H3K36me3 in *Kc* cells. In our description of H4K16Ac and H3K36me3 levels in S2 cells in Fig. 2 and Additional file 2, we used median values from these two different submissions. We obtained MSL-1 binding results from modENCODE submission ID 3293. These datasets can also be obtained from Gene Expression Omnibus (GEO, [77] with these accession IDs: GSE27805-6, GSE20797-9 and GSE32762. modENCODE study [51] provided smoothed log-intensity values between ChIP signal and the input signal, called *M* values, whose processed mean is shifted to 0. We used median *M* values within gene boundaries in describing MOF/MSL-1 binding or H4K16 acetylation in Fig. 2a–i and Fig. 3 (Additional file 3). MOF binding and H4K16 acetylation enriched/non-enriched regions in Fig. 4 directly followed peak-calls from the original study.

### Salivary gland expression profiles and ChIP-Seq results

We obtained microarray expression profiling and ChIP-Seq results from the third instar larva salivary glands for MOF binding and Histone H4K16 acetylation from [47]. The gene expression profiles were provided as GCRMA (GC Robust Multi-array Average, [78])-normalized signal intensities, and we used the top 99 percentiles of signals from non-*Drosophila* control probes as an expression cutoff. We demonstrated the median values from three replicates in Fig. 1c–e. The original results can be found from ArrayExpress [79] with accession ID of E-MEXP-3506. ChIP-Seq results for MOF binding and H4K16 acetylation, from the same study, can be accessed with ArrayExpress ID E-MTAB-911. In the result, the authors performed analysis with DESeq [80] to calculate log2 fold changes between ChIP and input samples for non-overlapping 25 bp windows across the genome. We used median values of such log2 fold changes within gene boundaries in describing the ChIP results in Fig. 2j–o.

### MSL entry sites

We used 150 CES that were characterized by ChIP-chip and ChIP-Seq studies [18] to generate a position weight matrix for MSL complex binding using MEME (Multiple EM for Motif Elicitation) suite version 4.11.2 [81]. We set the length of the motif to be 21 bp to match with the original CES study. Using the position weight matrix, we identified locations with MREs across the *Drosophila* genome release 5. We used FIMO 4.11.2 (Find Individual Motif Occurrences, [82] in this identification with *Expect* value (*E* value) threshold of 1.0e−05. In our description of MRE/CES occurrence in Fig. 2, we randomly shuffled positions of TADs on *X* chromosome genome using Bedtools 2.26.0 [83] while preserving the sizes of TADs. The results in Fig. 2r, s demonstrate overlap between such shuffled TADs and MRE/CES from 2000 randomizations.

### *S2* cell RNAi results for MSL knockdown and *roX* mutant larvae

We used *mof*, *msl*-*1*, *msl*-*3* knockdown results from a microarray study [47] (ArrayExpress E-MEXP-1505). For the estimation of gene expression changes, we used Robust Multi-array Average (RMA) [84] method for background adjustment and normalization and filtered out genes of which FPKM value is less than 1 from the *S2* cell RNA-Seq result [48]. We use R limma package version 3.28.21 [85] as in the official manual for our differential expression analysis. We obtained the microarray study of the *msl*-*2* knockdown data from [54]. We conducted the same data handling process as above. We also re-analyzed RNA-Seq results from [23] (GEO GSE16344). We used HISAT 2.0.4 [86] for the mapping of sequencing reads to *Drosophila* genome release 5. We used a parameter for unpaired sequencing (−U) in running HISAT. We measured gene-level read abundances with HTSeq 0.6.1 [86] with the default setting. From the counting result, we used polyA^+^ protein-coding genes that have more than 1 count per million mapped reads from any of the four samples (two controls and two RNAi) in our differential expression analysis. We performed differential expression analysis using DESeq 2 [87]. In Fig. 4, we demonstrated genes of which expression is more than 1 FPKM, which we also used to filter microarray results from MSL knockdown.

We re-analyzed the results from a previous study of mutant larvae that are null for *roX1* and *roX2* [88]. We performed the RMA normalization. The normalized signals had a bimodal distribution that is a mixture of two Gaussian distributions corresponding to signals from expressed genes versus that of experimental background and lowly expressed genes [89]. We generated a fitting model for the second distribution with the Expectation–Maximization method [90]. We took the top 99.9 percentile of it (= RMA 5.11) as the expression cutoff. We used R limma package for the differential expression analysis as described above.

## Additional files

**Additional file 1:** Gene ontology analysis results of the repressive genes. A full list of enrichment gene ontology terms from genes within the repressive TADs from FlyMine [76].

**Additional file 2:** *X*-linked genes within repressive TADs demonstrate lower H3K36me3 levels compared to non-repressive TAD genes. **(a)** Boxplots display normalized signal for H3K36me3 levels from *Kc* cells (top) and S2 cells (bottom). Signals from the LAD regions (gray) and non-LAD regions (white) were compared. **(b)** Comparisons between genes within Null and the other Hi-C domains. **(c)** Comparisons between genes within BLACK and the other DamID domains.

**Additional file 3:** A tabulated summary of repressive TAD information, gene expression levels, fold changes, ChIP signals used for study.

## Abbreviations

CES: Chromosome entry sites
ChIP: Chromatin immunoprecipitation
DCC: Dosage compensation complex
FPKM: Fragments Per Kilobase of transcript per Million mapped reads
GEO: Gene Expression Omnibus
GO: Gene ontology
H4K16Ac: Histone H4 lysine 16 acetylation
HAS: High-affinity sites
LAD: Lamina-associated domains
MLE: Maleless
modENCODE: Model organism ENcyclopedia of DNA Elements
MOF: Males absent on the first
MRE: MSL-recognition element
MSL: Male-specific lethal complex
NAR: Nucleoporin-associated region
NSL: Non-specific lethal
TAD: Topologically associated domain
