# Supplementary figures and images for: Non-canonical Drosophila X chromosome dosage compensation and repressive topologically associated domains
Source: Epigenetics Chromatin. 2018 Oct 24;11:62. doi: 10.1186/s13072-018-0232-y (PMC6199721; doi:10.1186/s13072-018-0232-y)

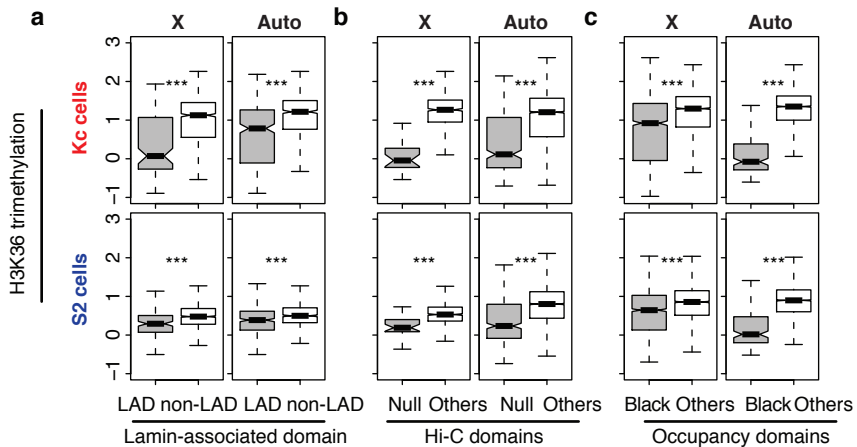

Supplement: Supplementary file 2 — Additional file 2: X-linked genes within repressive TADs demonstrate lower H3K36me3 levels compared to non-repressive TAD genes. (a) Boxplots display normalized signal for H3K36me3 levels from Kc cells (top) and S2 cells (bottom). Signals from the LAD regions (gray) and non-LAD regions (white) were compared. (b) Comparisons between genes within Null and the other Hi-C domains. (c) Comparisons between genes within BLACK and the other DamID domains. [file 13072_2018_232_MOESM2_ESM.pdf]
